# 

**Published:** 1829-01

**Authors:** 


### LONDON MEDICAL AND PHYSICAL JOURNAL.

EDITED BY

JOHN NORTH, ESQ. F.L.S.

MEMBER OF THE ROYAL COLLEGE OF SURGEONS,
AND OF THE MEDICAL AND CHIRURGICAL SOCIETY OF LONDON;

AND

JOHN WHATLEY, M.D. A.M.

MEMBER OF THE ARCADIA OF ROME;
MEMBER EXTRAORDINARY OF THE ROYAL MED. SOCIETY OF EDINBURGH;
MEMBER OF THE MEDICAL AND CHIRURGICAL SOCIETY OF LONDON;
AND OF THE MEDICAL SOCIETY OF LONDON.

(VOL. LXI.)

NEW SERIES, VOL. VI.

Et quoniam variant morbi, variabimus artes;
Mille mali species, mille salutis erunt.

LONDON:

JOHN SOUTER, 73, ST. PAUL'S CHURCH-YARD,

AND TO BE HAD OF ALL THE MEDICAL BOOKSELLERS.

1829.

45594

WI
LO31

J. AND C. ADLARD, PRINTERS,
BARTHOLOMEW CLOSE.

### MONTHLY LIST OF MEDICAL BOOKS.

[*Medical Works cannot be entered on this List except a copy be sent for the purpose; the titles of Books having frequently been transmitted to us, as published, which have not appeared for weeks, or even months, after.*]

A Manual on Midwifery; or, a Summary of the Science and Art of Obstetric Medicine; including the Anatomy, Physiology, Pathology, and Therapeutics, peculiar to Females; Treatment of Parturition, Puerperal and Infantile Diseases; and an Exposition of Obstetrico-legal Medicine. By MICHAEL RYAN, M.D. &c.—8vo. London, 1828.

A Practical Treatise on Parturition, comprising the attendant Circumstances and Diseases of the Pregnant and Puerperal States. By SAMUEL ASHWELL, Member of the Royal College of Surgeons, and of the Medico-Chirurgical Society of London. To which are appended two Papers, the one containing some Remarks on Abdominal Surgery, the other on Transfusion; presented by Dr. BLUNDELL, of Guy's Hospital.—8vo. London, 1828.

